# Retraction: Sentiment analysis of classical Chinese literature: An unsupervised deep learning model with BERT and graph attention networks

**DOI:** 10.1371/journal.pone.0357748

**Published:** 2026-09-08

**Authors:** The PLOS One Editors

The *PLOS One* Editors retract this article [1] due to concerns about:

- Compliance with PLOS policy on Manipulation of the Publication Process.
- The articles cited as References 6, 20, and 39 do not appear to support their respective corresponding cited statements.
- The article cited as Reference 37 was retracted after [1] was published.

All authors either did not respond directly or could not be reached.
